# Automated pupillometry and optic nerve sheath diameter ultrasound to define tuberculous meningitis disease severity and prognosis

**DOI:** 10.1016/j.jns.2023.120808

**Published:** 2023-09-13

**Authors:** Flora Casey, Hoang Minh Tu Van, Joseph Donovan, Ho Dang Trung Nghia, Pham Kieu Nguyet Oanh, C. Louise Thwaites, Nguyen Hoan Phu, Guy E. Thwaites

**Affiliations:** a London School of Hygiene and Tropical Medicine, Keppel St, London, UK; b Northern Adelaide Local Health Network, South Australia, Australia; c Oxford University Clinical Research Unit, Ho Chi Minh City, Viet Nam; d Hospital for Tropical Diseases, Ho Chi Minh City, Viet Nam; e Pham Ngoc Thach University of Medicine, Ho Chi Minh City, Viet Nam; f Centre for Tropical Medicine and Global Health, Nuffield Department of Medicine, University of Oxford, Oxford, UK; g School of Medicine, Vietnam National University of Ho Chi Minh City, Viet Nam

**Keywords:** Tuberculous meningitis, Intracranial pressure, Automated pupillometry, Optic nerve sheath diameter

## Abstract

**Background:**

Tuberculous meningitis (TBM) causes high mortality and morbidity, in part due to raised intra-cranial pressure (ICP). Automated pupillometry (NPi) and optic nerve sheath diameter (ONSD) are both low-cost, easy-to-use and non-invasive techniques that correlate with ICP and neurological status. However, it is uncertain how to apply these techniques in the management of TBM.

**Methods:**

We conducted a pilot study enrolling 20 adults with TBM in the Hospital for Tropical Diseases, Ho Chi Minh City, Vietnam. Our objective was to investigate the relationships between baseline and serial measurements of NPi and ONSD and disease severity and outcome. Serial NPi and ONSD were performed for 30 days, at discharge, and at 3-months, with measurements correlated with clinical progression and outcomes.

**Results:**

ONSD and NPi measurements had an inverse relationship. Higher ONSD and lower NPi values were associated with lower Glasgow coma score. Baseline NPi was a strong predictor 3-month outcome (median NPi 4.55, interquartile range 4.35–4.65 for good outcomes versus 2.60, IQR 0.65–3.95 for poor outcomes, *p* = 0.002). Pupil inequality (NPi ≥0.7) was also strongly associated with poor 3-month outcomes (*p* = 0.006). Individual participants’ serial NPi and ONSD were variable during initial treatment and correlated with clinical condition and outcome.

**Conclusion:**

Pupillometry and ONSD may be used to predict clinical deterioration and outcome from TBM. Future, larger studies are need explore the optimal timing of measurements and to define how they might be used to optimise treatments and improve outcomes from TBM.

## 1 Introduction

Tuberculous meningitis (TBM) is the most severe form of tuberculous, with death occurring in up to 50% of those affected [1–3]. Consistently poor outcomes from this devastating neurological infection can be attributed to the high frequency of neurological complications. These include hydrocephalus, cerebral infarction, tuberculomas and paradoxical reactions, all of which can lead to a common endpoint of raised intracranial pressure (ICP) [4–6].

The gold standard method of ICP monitoring requires insertion of an invasive neurological device in a neuro-intensive care unit setting. Insertion of intraventricular catheters, intraparenchymal pressure transducers, and subarachnoid bolts is expensive, and carries a risk of infection and bleeding [6]. These forms of ICP monitoring are often unavailable to individuals with TBM, who commonly reside in low- and middle-income countries with limited access to these techniques and facilities.

In the absence of invasive ICP monitoring, a variety of non-invasive methods of neurological monitoring are used to evaluate ICP and clinical status in patients with TBM. Glasgow coma score (GCS), fundoscopy and neuroimaging (computed tomography [CT] or magnetic resonance imaging [MRI]) each have limitations [7–12], such as lack of availability in all settings, poor correlation with ICP, and variability with other clinical circumstances including metabolic disturbances, sedation, or other medication use. Over recent years, automated pupillometry and optic nerve sheath diameter (ONSD) ultrasound have become recognised as promising non-invasive ICP monitoring tools. To date, the majority of evidence for their clinical use comes predominantly from outside the field of brain infection [13–17].

ONSD ultrasound measures the diameter of the optic nerve sheath. When ICP is elevated, elevations in the pressure of cerebrospinal fluid in the brain are transmitted to the sheath, which can swell in response. ONSD ultrasound has shown correlation with invasively measured ICP and neuroimaging in individual studies and meta-analyses [17–22]. In 107 adults with TBM in Vietnam, a higher ONSD value was associated with more severe TBM, and with abnormal neuroimaging [23]. In view of its low cost and non-invasive application, ONSD has great potential in monitoring and predicting outcome in patients with TBM.

Automated pupillometry is a non-invasive, portable and easy to use technique that records quantifiable data regarding pupil response to a light stimulus [24–26]. Pupil responsiveness to light is known to correlate with neurological condition and ICP, and pupillary response to a light source emitted by a simple pen torch has been a key part of basic neurological assessment for many years [27,28]. However, non-automated measurements of pupillary response are prone to error, being both user dependent and subjective [24,29]. The automated pupillometer emits a reproducible light stimulus towards the eye, and measures several variables: maximum and minimum pupil size, percentage change and speed of constriction and dilatation which gives a scaled value output termed the neurological pupil index (NPi), measured between values of 0 and 5 [26,30,31]. An NPi value ≥3 is considered normal, whereas a value of 0 would indicate a fixed, non-reactive pupil. A difference between the eyes of ≥0.7 is considered abnormal and a marker of pupil inequality [30,31]. Evidence supporting automated pupillometry use in neurological injury assessment has focused on traumatic brain injury and atraumatic intracranial haemorrhage. These data show NPi to have strong correlation with patient outcome measures, and with invasively measured ICP [13–15,24,28,32]. Additionally, medications commonly used in patients with neurological injury have limited effect on NPi [33]. Currently, there are no published studies describing automated pupillometry as a monitoring tool in brain infections, including TBM.

Early detection of elevated ICP in patients with TBM may allow essential therapies to reduce ICP to be administered before neurological injury becomes irreversible. We, therefore, conducted a pilot study to evaluate the role of automated pupillometry and ONSD ultrasound in adults with TBM. We sought to determine any correlation of NPi and ONSD with presenting TBM severity and with clinical outcome, and collected longitudinal data to understand how measured parameters – NPi and ONSD - fluctuate during mild and severe illnesses.

## 2 Methods

### 2.1 Study group

HIV-negative adult patients (≥18 yrs. old), with a suspected or confirmed diagnosis of TBM, with Medical Research Council Grade II or III [34] (i.e. moderate or severe disease) admitted to the Hospital for Tropical Diseases, Ho Chi Minh City, Vietnam, were eligible for enrolment into the study.

All participants enrolled in this pilot study were participants of the ‘LAST ACT trial’ (NCT03100786) – a leukotriene A4 hydrolase *(LTA4H)* genotype stratified, parallel group, randomised, double blind, placebo-controlled multi-centre phase III non-inferiority trial evaluating dexa-methasone versus placebo for 6–8 weeks in addition to standard anti-tuberculosis drugs in HIV-negative adults [35]. Patients and physicians remained blinded to treatment allocation throughout the study and the data analysis. The trial completed enrolment of 720 participants in March 2023 and will close, following completion of 1-year follow-up of all patients, in March 2024.

Patients with eye, facial or head trauma were excluded. The study had ethical approval by the HTD in Vietnam and through Oxford Tropical Research Ethics Committee (OxTREC). All participants or their legal representatives gave written consent to take part on the LAST ACT trial and the pupillometry sub-study.

### 2.2 Clinical data

Baseline clinical and demographic data were collected from participants including gender, age and medical comorbidities, presenting symptoms and neurological examination findings. Participants had daily GCS recorded during hospital admission. Routine blood test results (full blood count, urea, creatinine, sodium, glucose) and cerebrospinal fluid parameters, were recorded. TBM severity was recorded using the modified Medical Research Council severity grading [34].

Participants were categorised by their outcomes 3 months after study entry as ‘complete recovery’, ‘incomplete recovery’, ‘discharged in severe condition’ or ‘death’. Incomplete recovery referred to any lasting neurological sequelae such as reduced GCS, limb weakness or speech disturbance. Severe condition referred to those with incomplete recovery in addition to ongoing haemodynamic instability according to their vital signs.

### 2.3 Automated pupillometry and ONSD ultrasound

Automated pupillometry and ONSD ultrasound measurements were measured daily for 30 days (excluding public holidays and weekends), at hospital discharge and at a 3-month face-to-face follow up. Automated pupillometry and ONSD ultrasound measurements were performed by Dr. Hoang Minh Tu Van.

Automated pupillometry was performed in each eye by NeurOptics® Pupillometer NPi®-200, [36] using a unique patient barcode to store participants’ data electronically. Participants were positioned lying supine or up to 30° head tilt; once in position a flash of light stimulated the pupil and pupillary response was recorded by the automated pupillometer. Only a few seconds were required to complete the scan per eye. NPi was used for analysis.

ONSD ultrasound was performed in each eye as previously described [23]. Scans were performed of each eye using a linear probe and Venue Go (GE Healthcare). Three values were recorded per eye with the average of these readings used for analysis.

### 2.4 Treatment

All participants in this study received the standard anti-tuberculosis treatment of TBM, in accordance with local and national guidelines. 12 months of anti-tuberculosis chemotherapy were administered: rifampicin, isoniazid, ethambutol and pyrazinamide for the first two months and then rifampicin, isoniazid and ethambutol for the remaining 10 months. Any participants with suspected resistance, allergy or intolerance to any of the primary regimen had secondary regimens selected by the attending physicians. As participants of the LAST ACT trial, participants received blinded dexamethasone or placebo (for *LTA4H* CT- or CC-genotypes), or open label dexamethasone (for *LTA4H* TT-genotype) [35].

### 2.5 Statistical analysis

For this pilot, observational study we aimed to enrol at least 20 patients with TBM grade II or III at diagnosis. Prior to data analysis, the participants with incomplete recovery and those discharged in a severe condition who subsequently died were grouped together as poor outcomes given the small sample sizes in these groups. Those who had full recovery were classified as having good outcomes. A test for association was performed with Fisher’s exact test when the total number of observations was <20 or an individual cell count <5. Analysis between groups was performed with Wilcoxon rank-sum test. Correlation between continuous variables was performed using the Pearson correlation coefficient. Statistical analysis was performed using Stata (Stata/SE 17.0 [37]).

## 3 Results

### 3.1 Study group

Twenty participants were enrolled in the study between 30th June 2020 and 9th May 2021. The median age of the study group was 37.5 years (range 18–72 years) (Table 1). At enrolment (19/20) 95% participants had Grade II TBM, and (18/20) 90% had microbiologically confirmed TBM with cerebrospinal fluid positive for acid fast bacilli by Ziehl-Neelsen smear, or positive for *Mycobacterium tuberculosis* by Gen-eXpert MTB/RIF. The remaining two participants were considered by the treating clinician to have TBM based on consistent symptoms and cerebrospinal fluid findings and were treated with anti-tuberculosis chemotherapy. Including follow up, there were 407 days of data collection, encompassing 812 automated pupillometry readings and 774 ONSD ultrasound readings.

### 3.2 Outcomes

Ten participants had a complete recovery by time of discharge. One of these participants was lost to 3-month follow up due to COVID-19 pandemic restrictions. Of seven participants who were discharged with incomplete recovery, one subsequently improved and had completely recovered by 3-months, four remained without complete recovery by 3-months, and two of the participants were lost to 3-month follow up again due to COVID-19 pandemic restrictions. Two participants were discharged in a severe condition and died shortly after discharge.

### 3.3 Relationship between NPi and ONSD

**Fig. 1:**
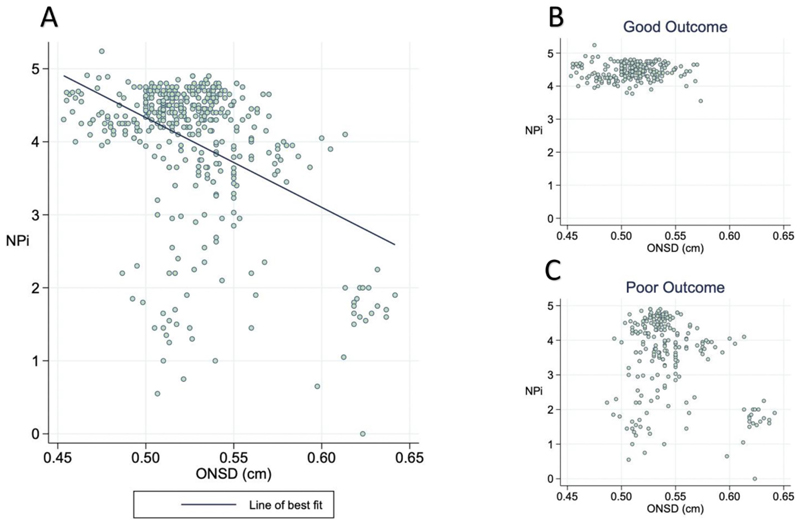
The relationship between NPi and ONSD. Graph A shows the relationship between NPi and ONSD with a line of best fit – Pearson correlation coefficient – 0.42, *p* < 0.001. Graphs B and C show the same data but split into the different outcome groups. Each participant contributes between 10 and 22 points. Abbreviations: ONSD = optic nerve sheath diameter; NPi = pupillometry.

An inverse relationship was found between NPi and ONSD (correlation coefficient – 0.42, *p < 0.001*) (Fig. 1A**)**, with higher ONSD readings associated with lower NPi values. Fig. 1B and C illustrate how individuals with good outcomes had normal NPi (>3) throughout, and had ONSD values <0.58 cm. However, in individuals with poor outcomes NPi and ONSD values were more variable with NPi range between 0 and 5 and ONSD range between 0.47 cm – 0.64 cm.

### 3.4 Relationship between GCS with NPi and ONSD

**Fig. 2:**
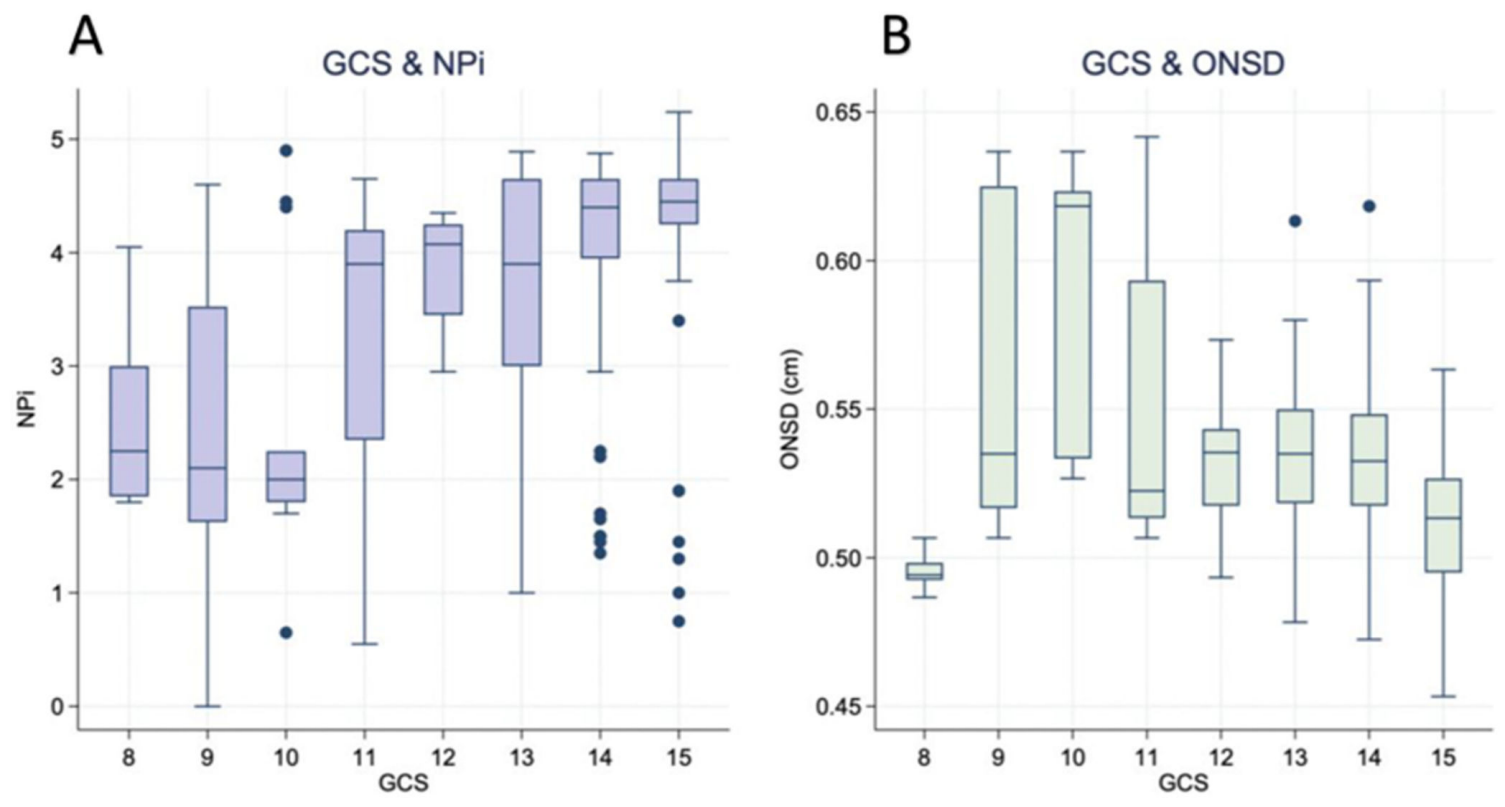
The relationships between NPi and ONSD, and GCS. Graphs demonstrating the relationship between GCS & NPi (A) and GCS & ONSD (B). The horizontal line within the box represents the median value, the lower line of the box represents the first quartile and the upper line the third quartile. The vertical lines above and below represent up to 1.5× the interquartile range. The dots outside the box represent outliers that don’t fall into the previous values. Abbreviations: GCS = Glasgow coma score; ONSD = optic nerve sheath diameter; NPi = pupillometry.

The relationships of NPi and ONSD with GCS are shown in Fig. 2. All participants had GCS between 8 and 15 during the study. Lower GCS was associated with lower NPi (correlation coefficient 0.56, *p < 0.001*) and with higher ONSD (correlation coefficient – 0.45, *p < 0.001*).

### 3.5 NPi trends and association with outcomes and pupil inequality

**Fig. 3:**
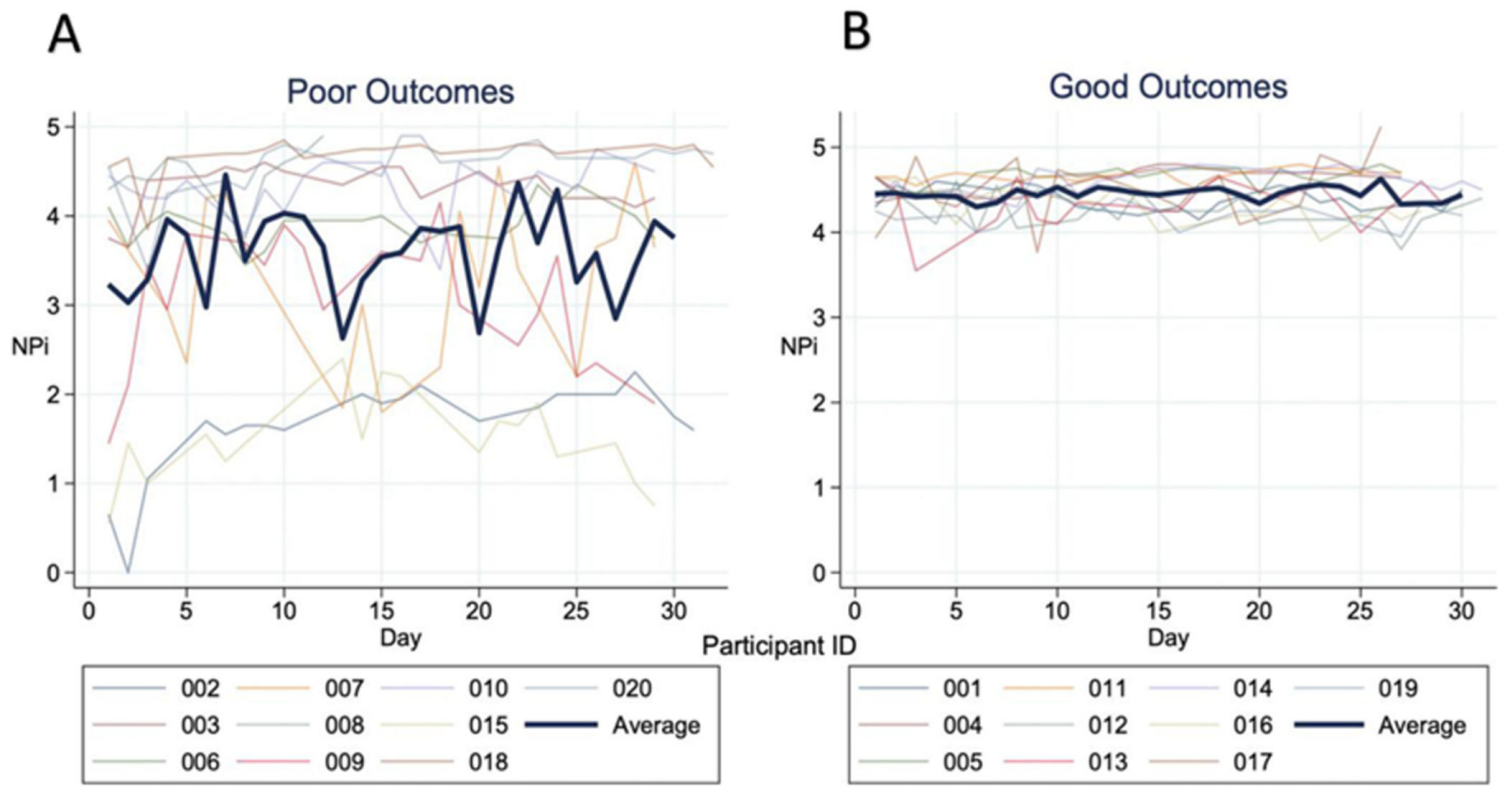
NPi over time by outcomes. Graph A showing the NPi of individual participants with poor outcomes over 30 days. Graph B showing the NPi of individual participants with good outcomes over 30 days. In both graphs the bold line is the average within that outcome group. Abbreviations: NPi = pupillometry.

Fig. 3 illustrates the difference in NPi throughout admission depending on outcomes at discharge. Individuals with poor outcomes had wider variation in NPi, whereas individuals with good outcomes had a more stable NPi which remained >3 throughout. Individuals with poor clinical outcomes had lower median NPi at admission; the median NPi was 4.55 (interquartile range [IQR] 4.35–4.65) in the good outcome group and 2.60 (IQR 0.65–3.95) in the poor outcome group *(p = 0.002)* (Table 2). Table 3 shows analysis of participants based on difference between eyes NPi. Difference in the NPi between eyes of ≥0.70 was considered relevant and a marker of pupil inequality. Eight participants were classified as having unequal pupils for at least one day during hospital admission. Unequal pupils at any point during admission was associated with worse outcomes at discharge (*p* = *0.006*) and at 3 month follow up *(p* = *0.007*).

### 3.6 ONSD trends and association with outcome

**Fig. 4:**
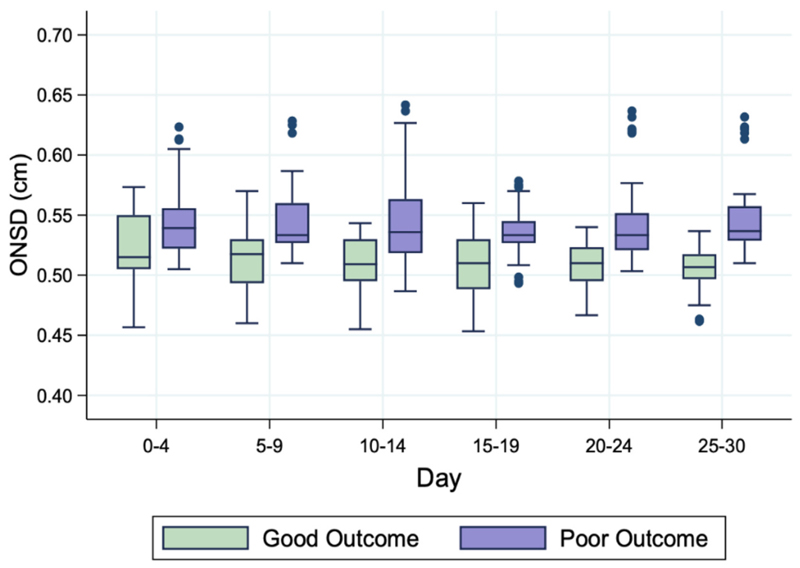
ONSD over time by outcomes. Box graph showing the ONSD over time stratified by outcomes. The data was grouped into 5-day periods. The horizontal line within the box represents the median value, the lower line of the box represents the first quartile and the upper line the third quartile. The vertical lines above and below represent up to 1.5× the interquartile range. The dots outside the box represent outliers that don’t fall into the previous values. Abbreviations: ONSD = optic nerve sheath diameter.

During admission, individuals with good outcomes had consistently lower ONSD values and individuals with poor outcomes had higher ONSD readings (Fig. 4). The median ONSD at admission was 0.51 cm (IQR 0.51–0.56) for those who were classified as good outcome at discharge and 0.54 cm (IQR 0.53–0.56) for those with a poor outcome.

## 4 Discussion

Our study demonstrates the potential utility of automated pupillometry and ONSD in adults with TBM. Baseline and subsequent NPi variation and pupil inequality were all strongly associated with poor clinical outcomes. NPi and ONSD showed good correlation in a side-by-side comparison. Serial ONSD measurements were more predictive of outcome than a single baseline measurement, supporting the findings of an earlier study [23].

This is the first study to investigate pupillometry for patients with TBM. There was a clear difference between those with good or poor outcomes and their NPi throughout admission. This suggests that even when the NPi remains above the normal threshold (≥3) any fluctuations may also be clinically significant. Additionally, when looking at individual participants it appears NPi change may precede a fall in GCS, suggesting NPi could be used as an early marker of clinical deterioration. Larger studies are required to define the optimal timing of the pupillometry and to determine its role in defining treatment.

Our study has some limitations. A larger sample size would have enabled further stratification of outcomes and increased power in analyses. Also, we only enrolled patients with moderate to severe diseases (MRC grade II or III) because we wanted to enrich the study with patients at most risk of deterioration and poor outcomes. However, the findings may not be generalisable to those with mild disease. Lastly, we did not compare the data from NPi or ONSD with the gold standard for ICP monitoring (invasive devices).

A strength of our study was the amount of longitudinal pupillometry and ONSD data collected. The study was carried out in a specialist hospital in Vietnam (HTD) with a long track record of TBM studies. The majority of both pupillometry and ONSD measurements were completed as scheduled, supporting the feasibility of these tools in a clinical setting – both ease of use by those performing the measurements and the compliance of the patients.

In conclusion, automated pupillometry and ONSD measurements were strongly associated with clinical deterioration and 3-month outcomes from TBM. There was an inverse relationship between NPi and ONSD and both had strong links with GCS. Future, larger studies are needed to strengthen the evidence for their use, investigating the optimal timing of the measurements and how they might influence treatment.

## Supplementary Material

Appendix A. Supplementary data

## Figures and Tables

**Table 1:** Baseline characteristics and demographic data of the participants at enrolment.

Variable	Frequency (total *n* = 20)
Gender	
Male	12 (60%)
Female	8 (40%)
HIV status	
Negative	20 (100%)
Positive	0 (0%)
TBM status	
Confirmed	18 (90%)
Suspected	2 (10%)
TBM MRC grade at presentation	
II	19 (95%)
III	1 (5%)
Neurological signs/symptoms at presentation	
Headache	20 (100%)
Fever (≥ 37.5degrees)	20 (100%)
Neck stiffness	18 (90%)
Cranial nerve palsy	9 (45%)
Irritability	7 (35%)
Lethargy	6 (30%)
Paraplegia	5 (25%)
Vomiting	3 (15%)
Hemiplegia	3 (15%)
Tetraplegia	2 (10%
Seizure	0 (0%)

Abbreviations: HIV = human immunodeficiency virus; TBM = tuberculous meningitis.

**Table 2:** Baseline ONSD & NPi results stratified by outcome.

Day 1 Variable	Outcome at discharge		*P value*
	Good *(n* = 10)	Poor (*n* = 9)	
Median ONSD (cm) (IQR)	0.51 (0.51–0.56)	0.54 (0.53–0.56)	*0.19*
Median NPi (IQR)	4.53 (4.30–4.65)	4.03 (1.45–4.45)	*0.020*
Day 1 Variable	Outcome at 3 months		*P value*
	Good (n = 10)	Poor (*n* = 6)	
Median ONSD (cm) (IQR)	0.53 (0.51–0.56)	0.54 (0.52–0.60)	*0.29*
Median NPi (IQR)	4.55 (4.35–4.65)	2.60 (0.65–3.95)	*0.002*

*P* values calculated using Wilcoxon rank sum test. Interquartile ranges displayed in brackets after values. Poor outcomes are those who had either incomplete recovery or those who were discharged in severe condition who subsequently died. Good outcome refers to those who had full recovery. Abbreviations: ONSD = optic nerve sheath diameter; NPi = pupillometry; IQR = Interquartile range.

**Table 3:** NPi difference between eyes and outcomes.

NPi difference between left and right eye ≥0.7 at any point	Outcome at discharge		*P* *value*
	Poor	Good	
Yes	7 (87.5%)	1 (12.5%)	*0.006*
No	2 (18%)	9 (82%)	
Total	9 (47%)	10 (53%)	
NPi difference between left and right eye ≥0.7 at any point	Outcome at 3 months		*P* *value*
	Poor	Good	
Yes	6 (75%)	2 (25%)	*0.007*
No	0 (0%)	8 (100%)	
Total	6 (37.5%)	10 (62.5%)	

Abbreviations: NPi = pupillometry.
