# Supplementary material for: Automated pupillometry and optic nerve sheath diameter ultrasound to define tuberculous meningitis disease severity and prognosis
Source: J Neurol Sci. Author manuscript; Available in PMC 2025 Apr 29. (PMC7617622; doi:10.1016/j.jns.2023.120808)
Supplement: Appendix A. Supplementary data [file EMS204245-supplement-Appendix_A__Supplementary_data.docx]

# **Supplementary appendices**

#
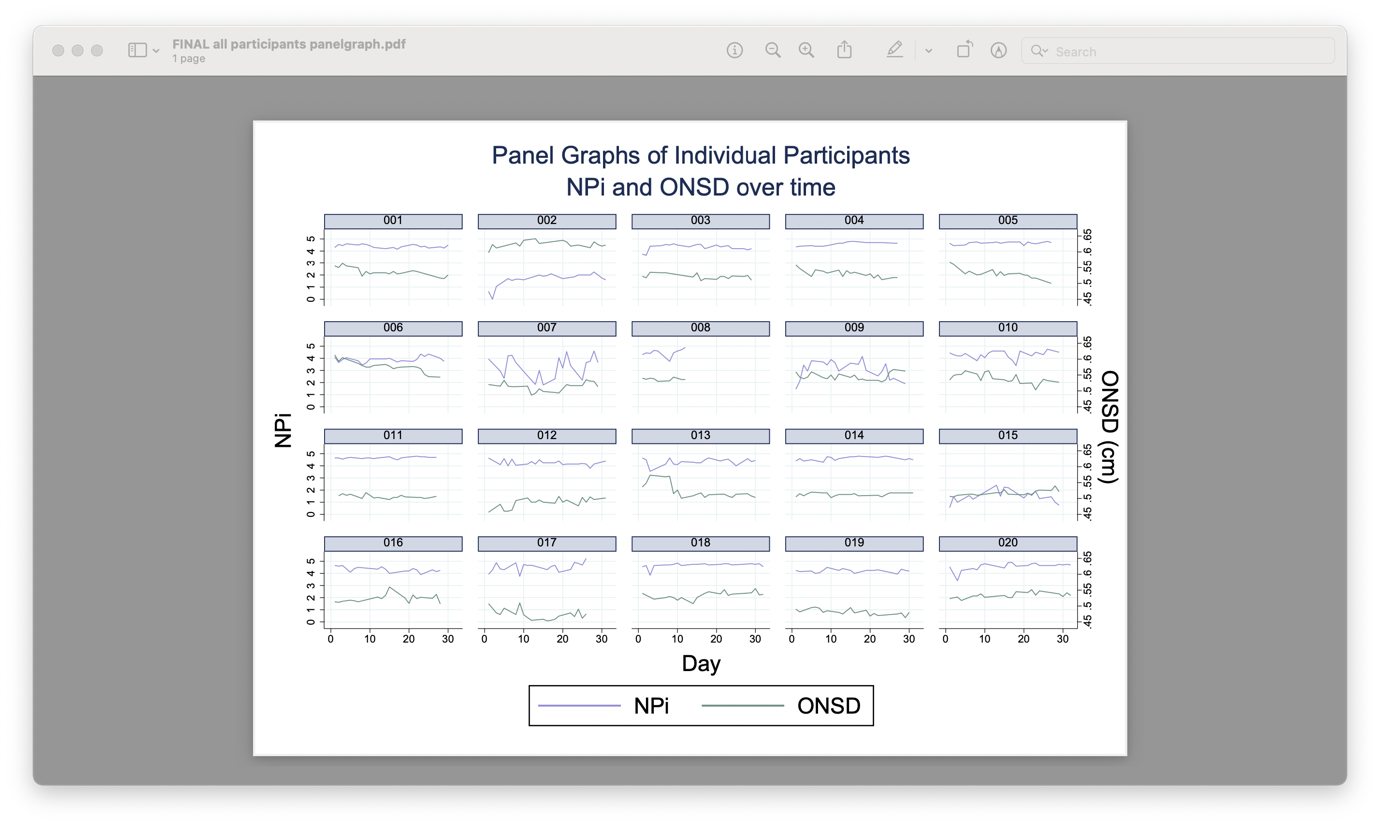
**Appendix figure 1: Individual participants trends in NPi and ONSD**

Appendix figure 1: Panel graphs of each individual participant with NPi on the right axis and ONSD on the left axis, over time on the y axis demonstrating individual trends. Abbreviations: NPi = pupillometry; ONSD = optic nerve sheath diameter.

**Appendix figure 2: Scanning matrix**

| Participant ID | Day | | | | | | | | | | | | | | | | | | | | | | | | | | | | | |
| --- | --- | --- | --- | --- | --- | --- | --- | --- | --- | --- | --- | --- | --- | --- | --- | --- | --- | --- | --- | --- | --- | --- | --- | --- | --- | --- | --- | --- | --- | --- |
|  | 1 | 2 | 3 | 4 | 5 | 6 | 7 | 8 | 9 | 10 | 11 | 12 | 13 | 14 | 15 | 16 | 17 | 18 | 19 | 20 | 21 | 22 | 23 | 24 | 25 | 26 | 27 | 28 | 29 | 30 - 32 |
| 001 |  |  |  |  |  |  |  |  |  |  |  |  |  |  |  |  |  |  |  |  |  |  |  |  |  |  |  |  |  |  |
| 002 |  |  |  |  |  |  |  |  |  |  |  |  |  |  |  |  |  |  |  |  |  |  |  |  |  |  |  |  |  |  |
| 003 |  |  |  |  |  |  |  |  |  |  |  |  |  |  |  |  |  |  |  |  |  |  |  |  |  |  |  |  |  |  |
| 004 |  |  |  |  |  |  |  |  |  |  |  |  |  |  |  |  |  |  |  |  |  |  |  |  |  |  |  |  |  |  |
| 005 |  |  |  |  |  |  |  |  |  |  |  |  |  |  |  |  |  |  |  |  |  |  |  |  |  |  |  |  |  |  |
| 006 |  |  |  |  |  |  |  |  |  |  |  |  |  |  |  |  |  |  |  |  |  |  |  |  |  |  |  |  |  |  |
| 007 |  |  |  |  |  |  |  |  |  |  |  |  |  |  |  |  |  |  |  |  |  |  |  |  |  |  |  |  |  |  |
| 008 |  |  |  |  |  |  |  |  |  |  |  |  |  |  |  | | | | | | | | | | | | | | | |
| 009 |  |  |  |  |  |  |  |  |  |  |  |  |  |  |  |  |  |  |  |  |  |  |  |  |  |  |  |  |  |  |
| 010 |  |  |  |  |  |  |  |  |  |  |  |  |  |  |  |  |  |  |  |  |  |  |  |  |  |  |  |  |  |  |
| 011 |  |  |  |  |  |  |  |  |  |  |  |  |  |  |  |  |  |  |  |  |  |  |  |  |  |  |  |  |  |  |
| 012 |  |  |  |  |  |  |  |  |  |  |  |  |  |  |  |  |  |  |  |  |  |  |  |  |  |  |  |  |  |  |
| 013 |  |  |  |  |  |  |  |  |  |  |  |  |  |  |  |  |  |  |  |  |  |  |  |  |  |  |  |  |  |  |
| 014 |  |  |  |  |  |  |  |  |  |  |  |  |  |  |  |  |  |  |  |  |  |  |  |  |  |  |  |  |  |  |
| 015 |  |  |  |  |  |  |  |  |  |  |  |  |  |  |  |  |  |  |  |  |  |  |  |  |  |  |  |  |  |  |
| 016 |  |  |  |  |  |  |  |  |  |  |  |  |  |  |  |  |  |  |  |  |  |  |  |  |  |  |  |  |  |  |
| 017 |  |  |  |  |  |  |  |  |  |  |  |  |  |  |  |  |  |  |  |  |  |  |  |  |  |  |  |  |  |  |
| 018 |  |  |  |  |  |  |  |  |  |  |  |  |  |  |  |  |  |  |  |  |  |  |  |  |  |  |  |  |  |  |
| 019 |  |  |  |  |  |  |  |  |  |  |  |  |  |  |  |  |  |  |  |  |  |  |  |  |  |  |  |  |  |  |
| 020 |  |  |  |  |  |  |  |  |  |  |  |  |  |  |  |  |  |  |  |  |  |  |  |  |  |  |  |  |  |  |

| **Key:** | |
| --- | --- |
|  | Weekend/public holiday |
|  | No NPi or ONSD recorded |
|  | Incomplete - Only NPi recorded |
|  | Both NPi and ONSD recorded |

Appendix figure 2: Scanning matrix depicting the actual scanning days for the participants. Dark blue represents both NPi and ONSD recorded. Light blue represents incomplete scanning (only NPi was performed) and orange represents a day when neither the NPi nor ONSD were recorded. White represents weekends or public holiday. See key attached. Abbreviations: NPi = pupillometry; ONSD = optic nerve sheath diameter.

**Appendix figure 3: An illustration of optic nerve sheath diameter with normal (A) and raised (B) intracranial pressure**


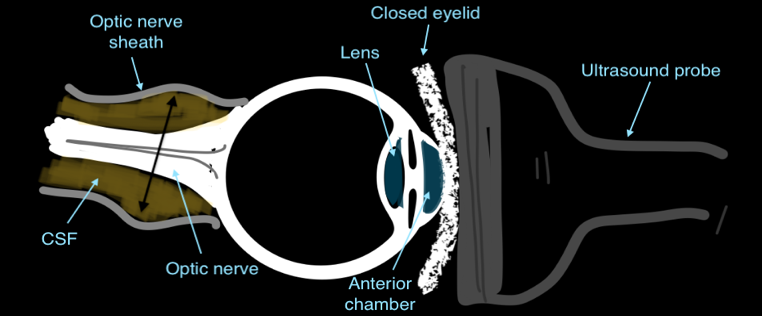

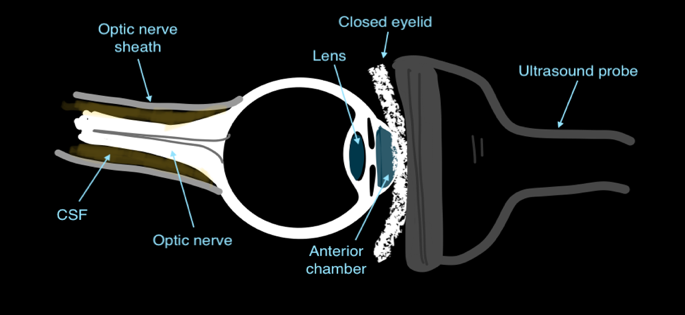


A

B

Appendix figure 3: Diagram depicting illustrations of the ultrasound images when measuring the ONSD. Panel A shows a normal ONSD and panel B shows an increased ONSD demonstrating how the optic nerve sheath swells in response to raised ICP. Abbreviations: CSF = cerebrospinal fluid; ICP = intracranial pressure; ONSD = optic nerve sheath diameter.
